# Neuron–tumor communication as a target for neuromodulatory drug delivery in cancer

**DOI:** 10.3389/fonc.2026.1929593

**Published:** 2026-10-30

**Authors:** Muhammad Esa, Tawseef Ahmad, Muhammad Khan, Asad Ur Rahman

**Affiliations:** 1 Drug Delivery System Excellence Center, Department of Pharmaceutical Technology, Faculty of Pharmaceutical Sciences, Prince of Songkla University, Hat Yai, Songkhla, Thailand; 2 Department of Clinical Pharmacy, Faculty of Pharmaceutical Sciences, Prince of Songkla University, Hat-Yai, Songkhla, Thailand; 3 Department of Pharmacology, Faculty of Pharmacy, Federal Urdu University of Arts, Sciences, and Technology, Karachi, Pakistan; 4 Chakri Naruebodindra Medical Institute, Faculty of Medicine Ramathibodi Hospital, Mahidol University, Samut Prakan, Thailand

**Keywords:** cancer neuroscience, drug delivery systems, nervous system, neural signaling, tumor innervation, tumor microenvironment

## Abstract

**Figure:**
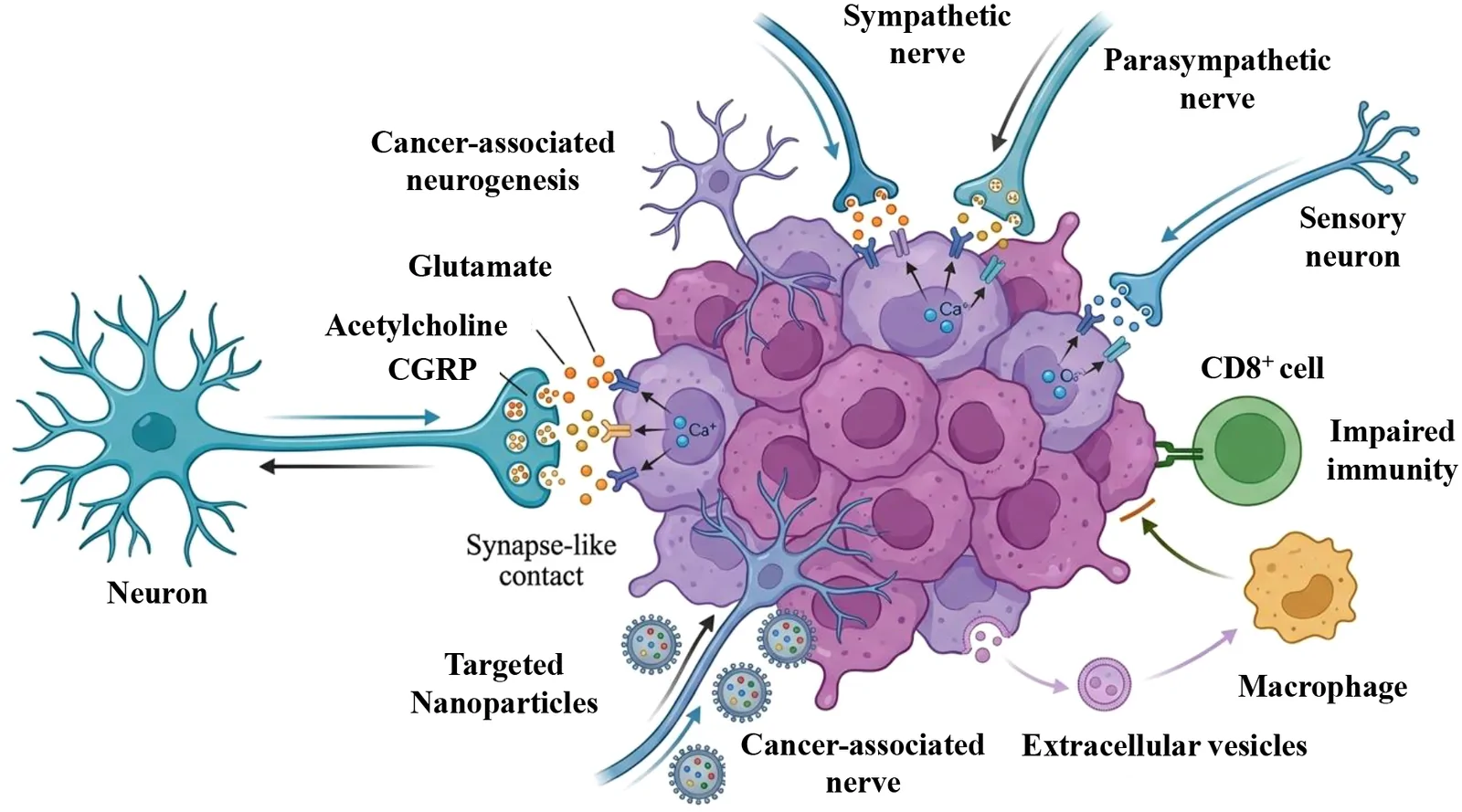

## 1 Introduction

The presence of nerves within tumors was largely unexplored in the early 19^th^ and 20^th^ century, with contemporary pathology texts generally considering tumors to be devoid of nerves. However, observations of tumor-associated pain and intratumoral nerve fibers challenged this view, prompting questions about their role in neoplastic tissue. As early as 1897, Young Y. reported nerves in various non-neural tumors, including breast and cervical carcinomas, sarcomas, myxomas, and metastatic carcinomas, providing early histological evidence of neural elements within tumors (1). This seminal study raised an important question of whether intratumoral nerves represented pre-existing neural structures incorporated during tumor growth or reflected active neural changes associated with neoplastic development.

To address this question, investigations in the late 1920s and early 1930s began to explore the origin and biological significance of these intratumoral nerves. In 1928, Herzog E. investigated the presence of nerve fibers in 100 neoplasms and proposed that intratumoral nerves represented pre-existing nerves incorporated into infiltrating neoplastic tissue. His observations suggested that the distribution of nerves was related to the pattern of tumor invasion, supporting the view that their presence could result from the incorporation of normal neural structures during tumor growth (2). In 1929, Oertel H. proposed a different interpretation, proposing that human carcinomas possessed an intrinsic neural organization, with nerve fibers closely associated with the tumor stroma, blood vessels, and parenchymal elements. This raised the possibility that the relationship between nerves and tumors might extend beyond passive entrapment (3). In 1930, Martynow W. further challenged the concept of simple nerve incorporation by suggesting that nerve fibers within carcinomas could be newly formed. He also described degenerative changes in these fibers, indicating that intratumoral nerves might undergo active remodeling in response to the tumor microenvironment (4).

In 1931, Oertel H., Nye H., and Thomlinson B. provided additional observations on nerve fibers within human tumors, further supporting the presence of an organized neural component within neoplastic tissue (5). In 1933, Ryrie synthesized these observations in his original investigation of the anatomical and physiological relationship between nerve fibers and neoplasms. He considered several possibilities, including the incorporation of pre-existing nerves into tumors, the formation of new nerve fibers within neoplastic tissue, and degenerative and regenerative changes resembling those observed after peripheral nerve injury. These observations raised the possibility of an active interaction between nerves and malignant tissue. Nevertheless, Ryrie G. concluded that the available evidence did not justify assuming a trophic influence of nerves on malignant cells (6).

Two studies published in 1982 and 1985 investigating neuroendocrine biomarkers also provided early biochemical evidence linking neural biology and cancer, demonstrating significantly elevated serum neuron-specific enolase (NSE) levels in patients with small-cell lung cancer (SCLC) (7, 8). In 1982, Carney et al. first reported that serum NSE was elevated in 69% of newly diagnosed patients with SCLC (n = 94) compared with controls, with a higher prevalence in extensive-stage disease (87%) than limited-stage disease (39%). Mean NSE levels were significantly higher in extensive-stage disease (59 ng/ml vs 13.8 ng/ml), increased further with metastatic burden (84% of patients with 1–2 sites and 100% of those with ≥3 sites), and showed a strong correlation with chemotherapy response. SCLC cell lines derived from patients also consistently expressed high levels of NSE (7). In a comparable study, Akoun et al. found that serum NSE levels were elevated in 65.1% of patients diagnosed with SCLC (n = 43), with significantly higher mean levels in extensive-stage disease (50 ng/ml) than in limited-stage disease (16 ng/ml), while levels were much lower in non-SCLC (11 ng/ml) and noncancerous lung disease (7 ng/ml). NSE closely correlated with tumor burden and showed dynamic changes during chemotherapy, with higher early levels (>100 ng/ml) associated with better treatment response (8). This mechanism was subsequently revealed in a 2022 study (**Figure 1**), which demonstrated that NSE interacts with and suppresses neuroblastoma suppressor of tumorigenicity 1 (NBL1), thereby relieving NBL1-mediated inhibition of BMP2 signaling. This interaction enhances BMP2–BMPR1A signaling and activates the BMP2/Smad/ID1 pathway, ultimately promoting malignant characteristics of SCLC (9).

**Figure 1:**
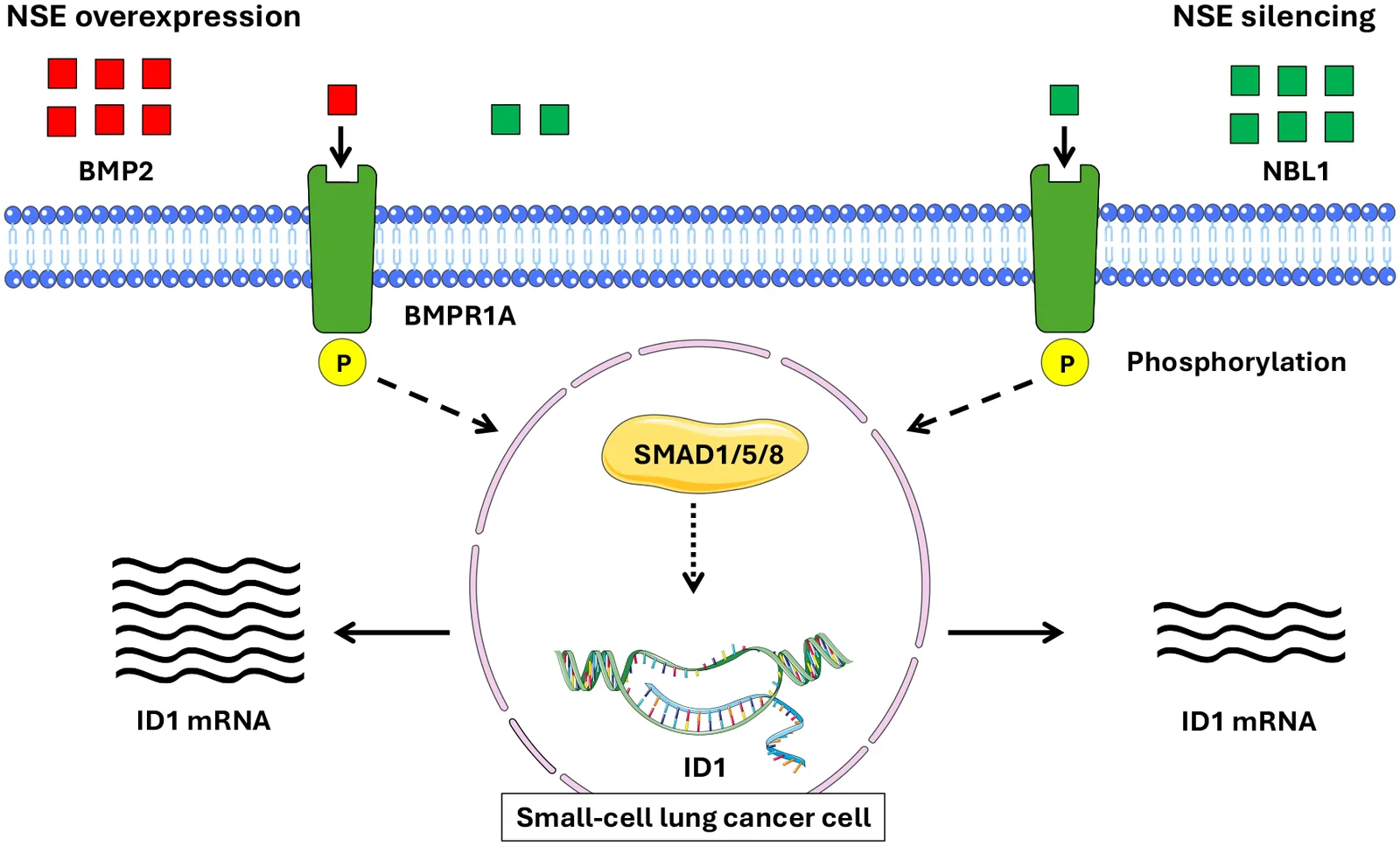
Proposed mechanism by which NSE promotes SCLC progression. (Left) NSE suppresses NBL1, allowing BMP2 to activate its receptor BMPR1A. (Right) This activates the SMAD1/5/8 pathway and increases ID1 activity, which promotes the growth and malignant behavior of SCLC cells. For a preliminary study of this mechanism, refer to (9).

Keeping track of these conceptual advances, a growing body of experimental evidence (detailed in section 2) have provided evidence of functional neuron-tumor interactions across multiple cancer types. Studies in human specimens have identified such interactions in squamous cell carcinoma (10), breast cancer (11), gastric cancer (12), pancreatic ductal adenocarcinoma (13), lung adenocarcinoma (14), prostate cancer (15), and diffuse midline glioma (16, 17). SCLC has been investigated using human specimens, *ex vivo* electrophysiological and co-culture models (18), and *in vivo* models (19), while glioblastoma (20, 21), glioma (22), diffuse midline glioma (16), gastric cancer (23), and breast-to-brain metastasis (24), have been studied primarily using *in vitro* and *in vivo* models. Evidence from these studies shows that neural signaling influences tumor growth, invasion, metastasis, and proliferation, while inhibition through pharmacological or genetic targeting of CHRM3, AMPARs, NMDARs/GRIN2B, GRIN2D, GABA receptors, NKCC1, CGRP, β-adrenergic signaling, and sensory or sympathetic neuron ablation has demonstrated therapeutic potential (12, 18, 22, 23, 25).

Several recent reviews have synthesized the rapidly expanding field of cancer neuroscience, encompassing interaction mechanisms, neuron–tumor synapses and pseudo-synaptic interfaces, neuroimmune and paracrine signaling, neural regulation of tumor progression and metastasis, and emerging therapeutic strategies targeting neural dependencies (26–29). Clinical translation has also been considered, with emphasis on drug repurposing, biomarker development, and clinical trial designs to advance neuroscience-informed cancer therapies (30). These reviews have already established the biological significance of neuron–tumor communication and highlighted its potential as a therapeutic target. However, the potential of neuron–tumor communication to inform and shape targeted drug-delivery strategies remains largely unexplored.

Following a careful review of the literature and registered clinical trials (Clinicaltrials.gov), we identified a growing but still limited effort to modulate neural signaling in cancer, with studies predominantly investigating β-adrenergic blockade, prostaglandin pathway inhibition, and local autonomic denervation. Clinical studies of propranolol (NCT01847001, NCT02420223, NCT00888797, NCT03384836, NCT00502684, NCT03838029, NCT04493489, NCT04682158, NCT05741164, NCT03919461, NCT05979818, NCT06839144, NCT07125391, and NCT07311915), etodolac (NCT00888797, NCT00502684, NCT03838029, NCT03919461, and NCT06839144), and botulinum toxin A (NCT01822210), administered predominantly systemically, highlight the therapeutic relevance of modulating neural signaling in cancer, although the clinical evidence remains preliminary and varies across approaches. In addition, glutamate-receptor antagonists, including riluzole (18), and NBQX (31), illustrate further pharmacological strategies for modulating tumor–neural communication, with varying levels of preclinical and clinical maturity. Propranolol-conjugated NP41-functionalised nanoparticles provide proof of concept for nerve-directed drug delivery in prostate cancer, validation across tumor types and systematic assessments of efficacy, selectivity, and neural safety remain necessary (32). Despite these contributions, the field of neuromodulation in cancer remains in its early stages, with key challenges in translating mechanistic insights into selective and clinically effective interventions. Therefore, in this Opinion, we propose the tumor–nerve interface as a potential target and discuss its implications for neuromodulatory drug delivery.

## 2 Experimental evidence supporting neural regulation of cancer

### 2.1 Neural regulation of tumor biology

Early experimental and clinical studies established that autonomic innervation can regulate tumor progression. In prostate cancer xenografts (PC-3), transgenic mouse models (Hi-Myc), and human prostate adenocarcinoma tissues (n = 43), sympathetic and parasympathetic nerves were found to exhibit distinct effects on tumor growth. While parasympathetic cholinergic nerve fibers penetrated tumors and encouraged invasion and metastasis, chemical or surgical sympathectomy and genetic ablation of stromal β2- and β3-adrenergic receptors significantly inhibited early tumor development. Analysis of human specimens further showed that high densities of sympathetic and parasympathetic nerve fibers were linked to aggressive clinicopathological features, such as increased levels of prostate-specific antigen, extracapsular extension, biochemical recurrence, and poor clinical outcomes (15). Similarly, in mouse models of gastric cancer (INS-GAS) and human gastric cancer specimens (n = 137), vagal cholinergic signaling was shown to drive tumorigenesis via muscarinic acetylcholine M3 receptor activation and Wnt pathway signaling. Surgical vagotomy, botulinum toxin type A (Botox), or pharmacological/genetic inhibition of the M3 receptor significantly reduced tumor growth, enhanced chemotherapy efficacy, and prolonged survival. Analysis of human specimens further showed that increased neural density correlated with advanced tumor stage and activated Wnt signaling, while clinical evaluation of patients with gastric stump cancer (n = 37) showed that vagotomy was associated with a reduced risk of gastric cancer (12).

Subsequent studies expanded this concept to sensory and sympathetic neural pathways across several tumor types (13, 33–36). In pancreatic ductal adenocarcinoma (PDAC), TRPV1^+^ sensory neurons promoted tumor development through neurogenic inflammation and reciprocal neuron–tumor signaling. In mouse models, neonatal capsaicin-mediated sensory neuron ablation significantly reduced pancreatic intraepithelial neoplasia formation, prevented perineural invasion and central nervous system inflammation, and prolonged survival (7.8 vs. 4.5 months, *p* = 0.001) (13). In a different study, sympathetic neural signaling also promoted PDAC progression through β-adrenergic mechanisms and was associated with increased intratumoral nerve density and brain-derived neurotrophic factor (BDNF) expression (36). In basal cell carcinoma, sensory nerves-maintained Hedgehog signaling in Gli1-expressing touch dome progenitor cells adjacent to Merkel cells. Surgical denervation significantly reduced touch dome-derived tumor formation without affecting hair follicle-derived lesions (33). In melanoma, sympathetic innervation was shown to promote tumor progression, as chemical sympathectomy with 6-hydroxydopamine significantly reduced tumor burden in B16-F10 models (0.29 vs. 1.02 g, *p* < 0.05). Denervation also altered the tumor microenvironment, increasing expression of apoptosis-related genes (Bcl-2 and caspase-3) and hypoxia-inducible factor-1α (34).

Beyond the effects of pre-existing nerves, evidence emerged that tumors can actively remodel and generate neural components. In human gastric and colorectal carcinomas, cancer stem cells differentiated into functional neuron-like cells expressing MAP2, TUJ, synaptic proteins (SV2A, Synapsin), and autonomic markers including TH^+^ sympathetic and VaChT^+^ parasympathetic neurons. In xenograft models, human neurons (NuMA^+^) originated from cancer stem cells and incorporated into the tumor ganglia and tissues, while silencing MAP2 significantly inhibited tumor growth *in vivo* (*p* < 0.01) and decreased neuronal production without affecting proliferation (37).

Consistent with these experimental findings, clinical studies have linked tumor-associated innervation with aggressive disease across multiple cancers. In upper aerodigestive tract squamous cell carcinoma, small peripheral nerves (<1 mm) were associated with increased perineural invasion, local recurrence, and poor survival (10). In breast cancer, increased tumor-associated nerve density and neurogenesis were associated with higher tumor grade, angiogenesis, and poor survival (11). Mechanistic studies further showed that SLIT2-mediated innervation and substance P/TACR1 signaling promoted tumor invasion, proliferation, circulating tumor cells, and lung metastasis in breast cancer (38). In lung adenocarcinoma, NGEF-associated sensory signaling through the Ephrin-A3/EphA2 pathway promoted tumor growth and progression and was associated with lymph node involvement and reduced overall survival (14).

### 2.2 Functional neuron–tumor communication

Beyond neural regulation of tumor biology, direct functional communication between neurons and tumor cells have been reported in multiple cancer types. In high-grade glioma, direct neuron–tumor synaptic communication was demonstrated, with presynaptic VGLUT1 and postsynaptic AMPA receptors colocalizing by more than 80% in 13.8–16.0% of tumor areas, indicating the presence of functional synapses. AMPA receptor antagonists and gap junction inhibitors inhibited the synaptic currents seen in about 31% of glioma cells. AMPA receptor blockage (perampanel or dominant-negative GluA2) markedly slowed tumor progression *in vivo*, neural activity increased tumor calcium signaling, invasion, and growth (22). GABAergic neuron–tumor communication has similarly been demonstrated in H3K27M-altered diffuse midline glioma, where functional neuron–glioma synapses and NKCC1-dependent chloride accumulation enabled GABA-induced depolarization and increased tumor cell proliferation and tumor burden (16). Similarly, glutamatergic, GABAergic, and cholinergic neuronal inputs increased neuron–tumor connectivity and neuronal activity, with cholinergic CHRM3 signaling promoting tumor invasion, proliferation, progression, and radiotherapy-associated resistance (20). Brain-wide neural integration was subsequently shown to activate ACh–CHRM3 signaling and downstream Ca²^+^-dependent transcriptional programs, increasing glioblastoma cell motility and invasion (21).

This functional paradigm was subsequently extended to metastatic disease, particularly brain metastases. In primary breast cancer samples (n = 1,100), expression of the N-methyl-D-aspartate receptor (NMDAR) pathway, particularly the GluN2B (GRIN2B) subunit, was strongly correlated with poor distant relapse-free survival. NMDAR signaling was significantly enhanced in brain metastases as compared to primary tumors or lung metastases using human and mouse breast-to-brain metastasis models (MDA231-BrM and TS1-BrM). Microscopic evaluations showed that metastatic cells positioned themselves adjacent to glutamatergic synapses, effectively replacing astrocytic processes and accessing neuron-derived glutamate. Functional co-culture tests found that direct neuron-tumor connections greatly increased metastatic cell proliferation, whereas genetic silencing of GRIN2B inhibited neuronal-induced growth. Importantly, inducible knockdown of GluN2B in intracardiac brain metastasis models significantly decreased brain metastatic burden (*p* < 0.05) and prolonged metastasis-free survival (*p* < 0.01) without affecting primary tumor growth or lung metastasis, indicating a brain-specific role for NMDAR signaling (24).

Functional neuron–tumor interfaces have also been identified outside the central nervous system. In PDAC, functional pseudo-synapses were identified between sensory neurons and cancer cells, with the N-methyl-D-aspartate receptor subunit 2D (GRIN2D) serving as a key mediator of neuron–tumor communication. Glutamate-mediated activation of GRIN2D promoted cancer cell migration, invasion, tumor growth, metastasis, and transforming growth factor alpha (TGFA)–epidermal growth factor receptor (EGFR)-dependent neuritogenesis, creating a feedforward loop that enhanced sensory innervation. Importantly, genetic deletion or pharmacological inhibition of GRIN2D significantly reduced tumor burden, metastasis, and peritumoral innervation while prolonging survival in orthotopic mouse models. Loss of GRIN2D similarly reduced tumor burden, metastasis, and peritumoral innervation while significantly prolonging survival in orthotopic mouse models. Ultrastructural investigations of human PDAC specimens demonstrated the existence of numerous neuron-cancer pseudo-synapses with presynaptic glutamate-releasing machinery and postsynaptic receptor complexes (25). Similarly, in gastric cancer, CGRP^+^ peptidergic sensory neurons formed functional circuits with tumor cells. Chemogenetic or optogenetic neuronal activation induced CGRP release and intracellular Ca²^+^ signaling in cancer cells, promoting tumor growth and metastasis, whereas CGRP inhibition or sensory neuron ablation reduced tumor progression and improved survival (23).

Further evidence of bona fide neuron–tumor synaptic communication has emerged from SCLC. In SCLC samples (n = 456), genomic analyses identified neuronal and synaptic signaling pathways as the major drivers of tumor progression. Using genetically engineered *Rb1^fl/fl^Trp53^fl/fl^* mouse models neuron–SCLC co-culture systems, *ex vivo* electrophysiology, and *in vivo* brain allograft models, the authors demonstrated that SCLC cells form functional glutamatergic synapses with host neurons. Rabies-virus tracing confirmed direct monosynaptic connectivity, while electrophysiological recordings verified active neurotransmission. Functional co-culture experiments showed that neuron–tumor synaptic interactions significantly enhanced SCLC proliferation (*p* < 0.05). Pharmacological inhibition of glutamatergic signaling with riluzole significantly delayed tumor progression and prolonged median overall survival from 54 to 71.5 days as monotherapy, and further extended survival by 21 days when combined with etoposide–cisplatin chemotherapy (18). **Table 1** summarizes experimental evidence from human specimens and *in vitro*/*in vivo* models, while the underlying mechanisms across cancer types are illustrated in **Figure 2**.

**Table 1:** Neural regulation and neuron–tumor communication across cancer types.

Cancer type	Neural component(s)	Neural communication/mechanism	Effect	Reference
Human specimens				
H3K27M-altered diffuse midline glioma	• GABAergic	• Active neuron–glioma synapses • NKCC1-mediated high intracellular Cl^-^	• GABA-induced depolarization • ↑ Tumor cell proliferation • ↑ Tumor burden	(17)
Diffuse midline glioma	• GABAergic	• ↑ Expression of GABA_A_ receptor (subunit α3, β3, γ2) • ↑ Postsynaptic machinery (*GPHN*, *NLGN2*)	• Tumors keep local GABAergic interneurons (GAD67^+^) intact in the surrounding tissue • Enriched in OPC-like malignant subpopulations across all primary patient tumors	(16)
Upper aerodigestive tract SCC	• Small peripheral nerves (<1 mm)	• ↑ Perineural invasion	• ↑ Local recurrence • Cervical lymph node metastasis • Disease-specific mortality • Poor survival	(10)
Breast cancer	• Tumor-associated nerves	• ↑ Neurogenesis/nerve density	• ↑ Tumor grade • ↑ Angiogenesis • Poor survival	(11)
Breast cancer	• Sensory neurons	• ↑ SLIT2-mediated innervation • ↑ SP/TACR1 signaling	• ↑ Tumor invasion and proliferation • ↑ Circulating tumor cells • ↑ Lung metastasis	(38)
Breast cancer with brain metastasis	• Glutamatergic	• ↑ *GRIN2B* & NMDAR pathway gene expression (enriched in basal-like/TNBC) • ↑ GluN2B phosphorylation (pGluN2B) in brain metastases vs. primary tumors	• ↑ Distant relapse and brain metastasis formation • ↑ High risk of recurrence across breast cancer subtypes • ↓ Distant relapse-free survival	(24)
Gastric cancer	• Vagal neurons	• ↑ Cholinergic/M3–Wnt/β-catenin signaling	• ↑ Tumor initiation and progression • ↑ Cancer stem-cell markers • ↑ Lymph node metastasis	(12)
Pancreatic ductal adenocarcinoma	• Sympathetic	• β-adrenergic signaling • ↑ BDNF-associated innervation	• ↑ Tumor progression • ↑ Intratumoral nerve density • ↑ BDNF expression	(36)
Pancreatic ductal adenocarcinoma	• Celiac ganglia and nerves invaded by cancer	• Enriched expression of GRIN2D, GRIN1, and postsynaptic PSD95 (DLG4) at sites of neural invasion • Concomitant enrichment of presynaptic VAMP1 and VGLU2 (SLC17A6) in invaded nerves	• ↑ Advanced T and N staging • ↑ Neural invasion severity • ↓ Overall survival • ↑ Disease recurrence	(25)
Lung adenocarcinoma	• Sensory neurons	• ↑ NGEF • ↑ Ephrin-A3/EphA2 signaling	• ↑ Tumor growth and progression • ↑ Lymph node involvement • ↓ Overall survival	(14)
In vitro/In vivo				
Glioblastoma	• Glutamatergic • GABAergicS • Cholinergic	• ↑ Neuron–tumor connectivity and neuronal activity • ↑ Cholinergic–CHRM3 signaling	• ↑ Tumor invasion, proliferation and progression • ↑ Radiotherapy-associated connectivity and resistance	(20)
Glioblastoma	• Cholinergic (glutamatergic and GABAergic)	• Brain-wide neural integration • ACh–CHRM3 signaling Ca²^+^/transcriptional activation	• ↑ Tumor cell motility • ↑ Invasion and progression • ↑ Survival following CHRM3 downregulation	(21)
Glioma	• Glutamatergic	• ↑ Direct neuron–glioma chemical synapses • ↑ AMPAR activation on glioma cells • Synaptic input occurs mainly along tumor microtubes	• ↑ Tumor microtube network activity • ↑ Tumor invasion and motility • ↑ Tumor proliferation and growth • ↓ Tumor progression with AMPAR inhibition	(22)
Diffuse midline glioma	• GABAergic	• Functional GABA_A_ neuron-to-glioma synapses • ↑ Cl^-^ efflux and membrane depolarization • Optogenetic interneuron stimulation (40 Hz) evokes picrotoxin-sensitive postsynaptic currents	• ↑ Tumor cell proliferation • ↑ Expression of growth gene (*Fos*) • ↓ Mouse survival (reversed by GABA_A_ antagonists or NKCC1 block with bumetanide)	(16)
High-grade glioma	• Glutamatergic	• ↑ AMPAR-mediated synaptic transmission • ↑ Activity-dependent extracellular K^+^ accumulation • ↑ Gap junction coupling via tube microtubes	• ↑ Tumor proliferation and progression • ↓ Mouse survival (reversed by AMPAR/gap junction block)	(22)
Breast-to-brain metastasis	• Glutamatergic • Presynaptic axons	• ↑ Formation of pseudo-tripartite synapses with glutamatergic neurons • ↑ NMDAR pathway activation	• ↑ NMDAR-dependent tumor proliferation • ↑ Brain metastatic colonization and growth • ↓ Brain metastasis-free survival (reversed by GRIN2B knockdown)	(24)
SCLC and SCLC brain metastasis	• Glutamatergic • GABAergic	• ↑ Vagal innervation • ↑ Neuronal activity leads to synaptic/paracrine signaling • ↑ Membrane depolarization/Ca²^+^ signaling	• ↑ Primary tumor development, progression and proliferation • ↑ Intracranial tumor growth	(19)
Gastric cancer	• CGRP^+^-peptidergic (sensory)	• ↑ Direct physical contact and functional circuit • ↑ Chemogenetic/optogenetic activation • ↑ CGRP release and intracellular Ca^2+^ flux	• ↑ Cytoplasmic calcium release in tumor • ↑ Tumor growth and metastasis • ↓ Mouse survival reversed by CGRP inhibitors (BIBN4096BS/Olcegepant at 1 mg/kg intraperitonially or sensory neuron ablation)	(23)
Pancreatic ductal adenocarcinoma	• Peripheral sensory neurons (DRG neurons, TRPV1+ nociceptors)	• ↑ Direct cancer–sensory neuron pseudo-synapses • ↑ Activation of CAMK4-CREB1 and E2F1-EZH2 signaling • ↑ GRIN2D expression and TGFA-EGFR signaling	• ↑ Cell migration, invasion, tumor growth, and metastasis • ↑ Peritumoral innervation and tumor-associated mechanical pain • ↓ Mouse survival (reversed by Grin2d KO/KD, GRIN2D antagonist UBP145, or TRPV1+ neuron ablation	(25)

ACh, acetylcholine; AMPAR, α-amino-3-hydroxy-5-methyl-4-isoxazolepropionic acid receptor; BDNF, brain-derived neurotrophic factor; CAMK4, calcium/calmodulin-dependent protein kinase IV; CGRP, calcitonin gene-related peptide; CHRM3, cholinergic receptor muscarinic 3; CREB1, cAMP response element-binding protein 1; DLG4, discs large MAGUK scaffold protein 4; DRG, dorsal root ganglion; E2F1, E2F transcription factor 1; EGFR, epidermal growth factor receptor; EZH2, enhancer of zeste homolog 2; GABA, gamma-aminobutyric acid; GABA_A_R, gamma-aminobutyric acid type A receptor; GAD67, glutamate decarboxylase 67; GPHN, gephyrin; GRIN1, glutamate ionotropic receptor NMDA type subunit 1; GRIN2D, glutamate ionotropic receptor NMDA type subunit 2D; KD, knockdown; KO, knockout; LUAD, lung adenocarcinoma; NGEF, neuronal guanine nucleotide exchange factor; NLGN2, neuroligin-2; NMDAR, N-methyl-D-aspartate receptor; NKCC1, Na^+^-K^+^-Cl^-^ cotransporter 1; OPC, oligodendrocyte precursor cell; SCC, squamous cell carcinoma; SCLC, small-cell lung cancer; SP, substance P; TACR1, tachykinin receptor 1; TGFA, transforming growth factor alpha; TNBC, triple-negative breast cancer; TRPV1, transient receptor potential vanilloid 1; VAMP1, vesicle-associated membrane protein 1; VGLUT2, vesicular glutamate transporter 2.

**Figure 2:**
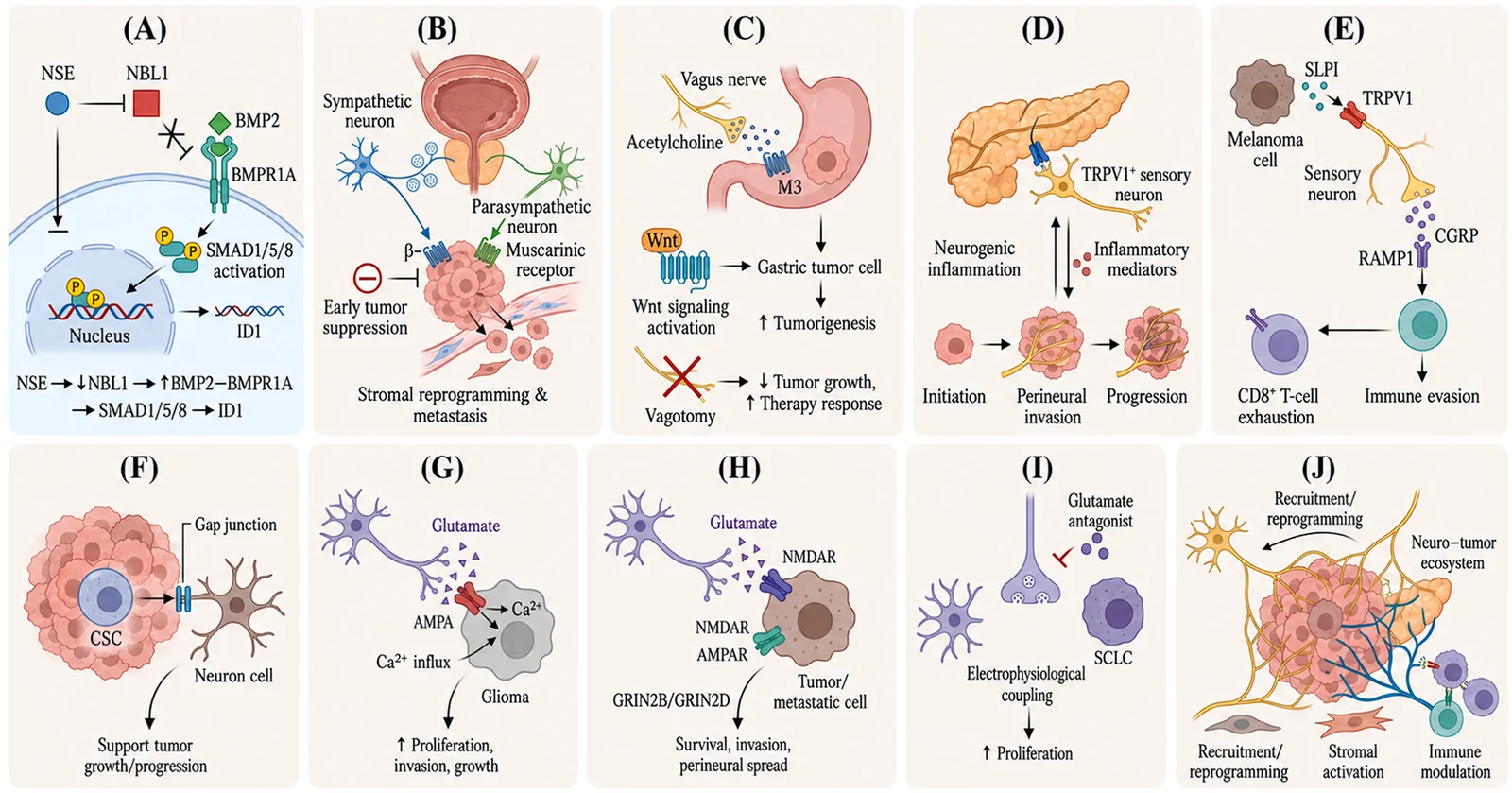
Mechanisms of neuron–tumor communication across cancer types. **(A)** In SCLC, NSE–NBL1 inhibition activates BMP2–BMPR1A–SMAD1/5/8–ID1 signaling, promoting stem-like properties. **(B)** In prostate cancer, β-adrenergic and cholinergic signaling promotes stromal reprogramming, invasion, and metastasis. **(C)** In gastric cancer, vagal acetylcholine activates M3–Wnt signaling to promote tumorigenesis. **(D)** In PDAC, TRPV1^+^ sensory neurons and inflammatory mediators support tumor initiation, perineural invasion, and progression. **(E)** In melanoma, tumor-derived SLPI activates TRPV1^+^ neurons and CGRP–RAMP1 signaling, promoting CD8^+^ T-cell exhaustion. **(F)** In CSC-driven tumors, cancer stem cells form neuron-like cells and gap-junction connections that support progression. **(G)** In glioma, neuronal glutamate activates AMPA receptors, increasing Ca²^+^ influx, proliferation, and invasion. **(H)** In brain metastasis and PDAC, tumor cells engage glutamatergic synapses and GRIN2B/GRIN2D-containing NMDA receptors to support survival and invasion. **(I)** In SCLC, glutamatergic neuron–tumor synapses promote proliferation, whereas glutamate antagonists reduce tumor growth. **(J)** In PDAC, neuronal recruitment and reprogramming contribute to a neuro–tumor ecosystem involving stromal and immune modulation. α-amino-3-hydroxy-5-methyl-4-isoxazolepropionic acid receptor (AMPAR); bone morphogenetic protein 2 (BMP2); bone morphogenetic protein receptor type 1A (BMPR1A); cancer stem cell (CSC); calcitonin gene-related peptide (CGRP); glutamate ionotropic receptor NMDA type subunit 2B (GRIN2B); glutamate ionotropic receptor NMDA type subunit 2D (GRIN2D); inhibitor of DNA binding 1 (ID1); muscarinic acetylcholine receptor M3 (M3); neuroblastoma suppressor of tumorigenicity 1 (NBL1); neuron-specific enolase (NSE); non-small cell lung cancer (NSCLC); N-methyl-D-aspartate receptor (NMDAR); receptor activity-modifying protein 1 (RAMP1); small cell lung cancer (SCLC); SMAD family members 1, 5, and 8 (SMAD1/5/8); transient receptor potential vanilloid 1 (TRPV1); and Wingless/Integrated signaling pathway (Wnt).

### 2.3 The neuro-immune axis

Neural regulation of tumors is mediated, in substantial part, through immune cells, making neuroimmune interactions central to both the therapeutic rationale and the challenges of drug delivery discussed in this article. Preclinical and clinical-correlative evidence highlights their role in tumor progression and treatment response (see **Figure 3**). For instance, activated and memory CD8^+^ T cells have been reported to predominantly express β2-adrenergic receptors. In preclinical mouse tumor models (BALB/c, C57BL/6, and 4T1), reducing β-adrenergic signaling with propranolol (200 μg in 100 μL of PBS, administered by intraperitoneal injection) or genetic deletion of β2-adrenergic receptors promoted an immunologically active tumor microenvironment, characterized by an increased frequency of effector-phenotype CD8^+^ T cells, reduced PD-1 expression, and an elevated effector CD8^+^ T-cell-to-regulatory T-cell ratio. These changes were associated with enhanced responses to anti-PD-1checkpoint blockade using anti-mouse PD-1 antibody (RMP1-14, 200 μg per injection, administered in 5–6 doses at 2–3-day intervals) (39). In melanoma (B16-OVA) and colon cancer (CT26.CL25), chronic stress-induced β-adrenergic signaling promoted T-cell exhaustion and impaired glycolysis and oxidative phosphorylation. Pharmacological blockade with propranolol (200 μg, administered intraperitoneally once daily) or genetic deletion of β2-adrenergic receptors reduced T-cell exhaustion, increased progenitor-exhausted T cells, and restored metabolic function. These interventions also increased CD28 expression and T-cell receptor signaling, enhancing anticancer effector functions, including IFN-γ, TNF-α, and IL-2 production (40). Consistent with these findings, pharmacological sympathetic denervation using 6-hydroxydopamine (three doses of 100 mg/kg each) induced a pro-inflammatory tumor microenvironment and enhanced the efficacy of nivolumab (immune checkpoint inhibitor) in PDAC models (41).

**Figure 3:**
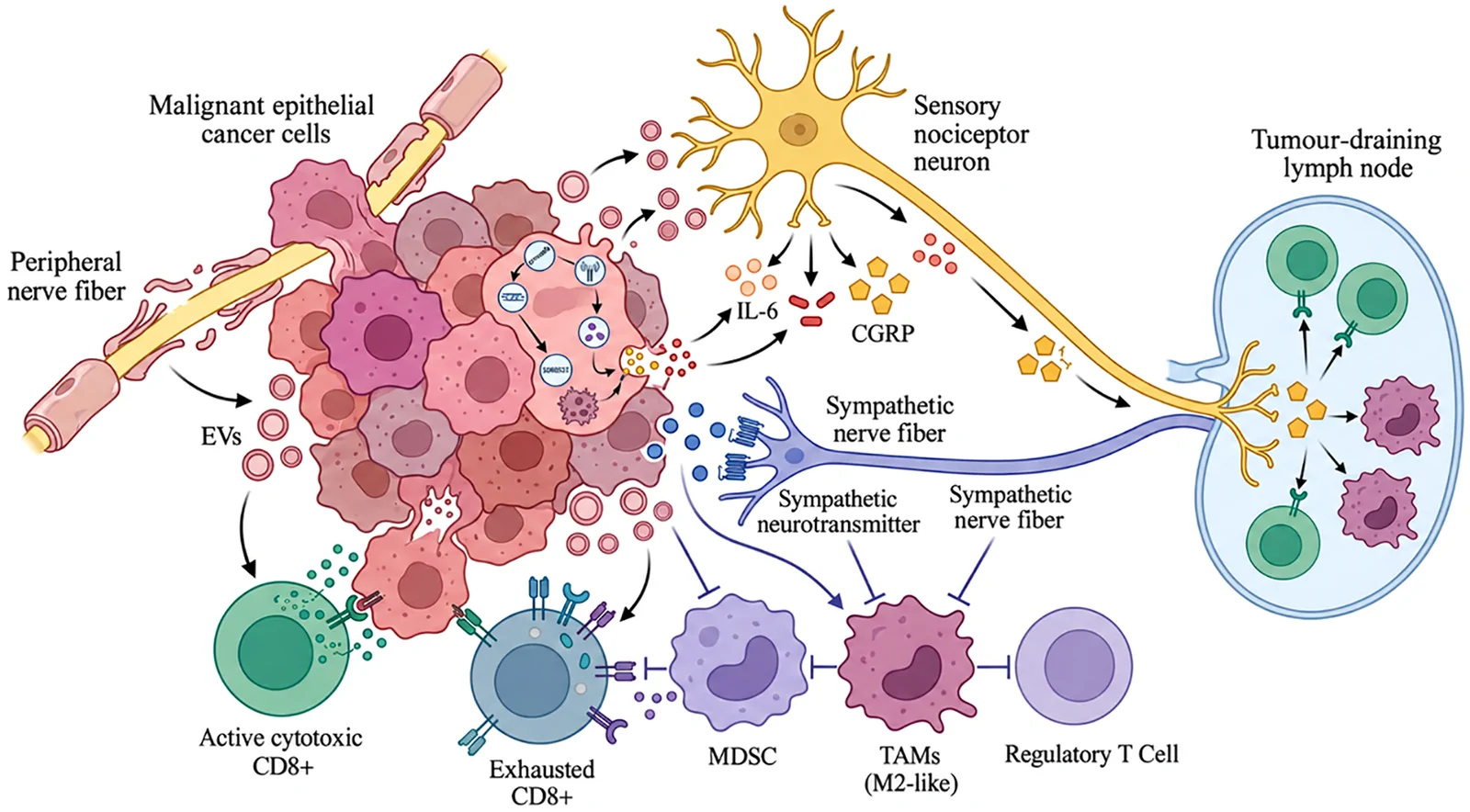
Neuroimmune mechanisms linking neural signaling to tumor progression and immunotherapy response. Sensory and sympathetic nerves regulate tumor-associated immune cells through neurotransmitters, cytokines and extracellular vesicles, influencing T-cell exhaustion, immunosuppressive cell populations and antitumor immunity. CD8^+^, cluster of differentiation 8-positive T cells; EVs, extracellular vesicles; IL-6, interleukin-6; M2, alternatively activated macrophages; MDSCs, myeloid-derived suppressor cells; TAMs, tumor-associated macrophages; T cells, T lymphocytes.

Sensory neurons also regulate antitumor immunity. In melanoma, TRPV1-lineage sensory neurons release calcitonin gene-related peptide (CGRP), which promotes CD8^+^ T-cell exhaustion through the receptor component RAMP1, impairing cytotoxic activity. Genetic ablation of TRPV1-lineage neurons, local pharmacological nociceptor silencing, or CGRP-receptor antagonism reduced immune-cell exhaustion and tumor growth, nearly tripling survival in mice. Notably, the study also found that higher RAMP1 expression in tumor-infiltrating CD8^+^ T cells in human melanoma was associated with greater exhaustion and poorer clinical outcomes (35). In head and neck squamous cell carcinoma and melanoma models, tumor-derived small extracellular vesicles reprogrammed nociceptors into an injury-like state (IL-6), promoting myeloid-derived suppressor cell expansion and dysfunctional, checkpoint-receptor-high CD8^+^ T cells. Neuronal ablation reduced myeloid-derived suppressor cell infiltration, while interactions between nociceptors and tumor-derived vesicles increased checkpoint-receptor expression on T cells, supporting tumor immune escape (42).

These circuits also extend beyond the primary tumor. In three mouse models of oral cancer, cancer cells under immune pressure released SLIT2 through an ATF4-dependent pathway, activating pain-sensing nerves. These nerves increased CGRP release in tumor-draining lymph nodes, reducing CCL5 levels and promoting tumor-supporting macrophages. This weakened the response to immunotherapy. Targeting nociceptors or disrupting the ATF4–SLIT2–CGRP pathway improved antitumor immunity and treatment responses (43). Similarly, cancer-induced nerve injury may also weaken antitumor immunity. In patients with cutaneous squamous cell carcinoma, melanoma, and gastric cancer, tumor invasion and damage to nerves were associated with poorer responses to cemiplimab (anti-PD-1) therapy. The injured nerves released IL-6 and type I interferons, causing chronic inflammation that promoted immunosuppression and CD8^+^ T-cell exhaustion as tumors grew. Blocking nerve activity, disrupting neuronal *Atf3* or *Ifnar1* signaling, or inhibiting the IL-6 receptor improved responses to anti-PD-1 therapy (44).

## 3 Clinical trials update

The available clinical trials (**Table 2**), retrieved on October 5, 2026, via ClinicalTrials.gov, span perioperative β-adrenergic and COX-2 inhibition, neoadjuvant β-blockade, hematopoietic stem cell transplantation, and direct modulation of tumor innervation. A phase III randomized, quadruple-masked trial (NCT00888797) evaluated perioperative propranolol plus etodolac in 400 patients undergoing curative-intent colorectal cancer resection, targeting surgery-induced immune suppression and recurrence. The primary endpoint was 3-year recurrence/metastasis, with postoperative immune and clinical outcomes as secondary endpoints, while related epidemiological data showed no clear association between β-blocker use and colorectal cancer-specific mortality (45). A phase II single-arm trial (NCT01847001) assessed propranolol with neoadjuvant chemotherapy in newly diagnosed breast cancer to modulate β-adrenergic signaling, tumor angiogenesis, and microvascular responses. Among 10 patients, adherence was 96%, 90% reached the target dose (80 mg/day), and 1 (10%) achieved a pathological complete response, supporting feasibility but not clinical efficacy because of the small, uncontrolled sample.

**Table 2:** Clinical trials evaluating pharmacological and therapeutic modulation of neural pathways in cancer, identified through ClinicalTrials.gov (accessed October 5, 2026).

Trial ID	Phase/design	Cancer type & setting	Neurol target	Intervention	Estimated enrollment	Status	Primary outcome/key result
NCT01847001	Phase II, single-arm, open-label, prospective interventional	Breast cancer, neoadjuvant chemotherapy before surgery	β-adrenergic signaling/stress pathway	Propranolol (20 mg BID, titrated to 80 mg/day) + paclitaxel + doxorubicin or cyclophosphamide)	20 (actual enrollment 10)	Completed (Oct 2019, results posted on ClinicalTrials.gov)	Propranolol adherence was 96% with 9/10 (90%) reaching the target 80 mg/day dose Pathologic complete response occurred in 1/10 (10%)
NCT02420223	Phase II, randomized, open-label, parallel-group pilot RCT	Multiple myeloma, autologous hematopoietic stem cell transplantation (HCT)	β-adrenergic signaling/stress-mediated gene expression	Propranolol (20 mg BID, titrated to 40 mg BID), started 7 days before HCT and continued to 28 days post-HCT	40 (actual enrollment 25)	Completed (May 2021, results posted on ClinicalTrials.gov)	Change in β-adrenergically mediated gene expression at 4 weeks post-HCT
NCT01822210	Phase II, single-arm, open-label pilot study	Gastric adenocarcinoma, unresectable/inoperable	Gastric autonomic innervation	Botulinum toxin A (100 units, local injection to the tumor)	10 (actual enrollment 7)	Completed (May 2016, results posted on ClinicalTrials.gov)	Change in tumor volume by CT over 8–20 weeks
NCT00888797	Phase III, multicenter, randomized, quadruple-masked RCT	Colorectal cancer, curative-intent resection	β-adrenergic signaling	Propranolol (20 mg BID) × 5 days pre-op → 80 mg BID on surgery day → 40 mg BID × 1 week → 20 mg BID × 1 week Etodolac (800 mg BID throughout 20-day intervention)	400	Unknown* Results published in 2013	3-year cancer recurrence/metastasis Postoperative immune suppression and morbidity/mortality as secondary outcomes No clear association with CRC-specific mortality (OR 0.89, 95% CI 0.78–1.02)
NCT03384836	Phase Ib/II, open-label, single-arm, dose-escalation followed by efficacy phase	Unresectable stage IIIC–IV melanoma, systemic treatment	β-adrenergic signaling/stress pathway	Propranolol + pembrolizumab	47	Suspended (interim analysis)	Phase Ib: dose-limiting toxicities Phase II: overall response rate
NCT00502684	Phase (NA), randomized, double-blind, placebo-controlled, interventional	Breast cancer, perioperative	β-adrenergic signaling	Propranolol (10 mg QID) Etodolac (400 mg BID)	148 patients across 4 RCTs: breast (38), CRC (34 + 46), pancreatic (30)	Unknown* Results published in 2026	Generally safe with no significant major adverse events CRC 8-year DFS improved
NCT03838029	Phase II, randomized, double-blind, placebo-controlled	Pancreatic cancer, curative-intent surgery	β-adrenergic + COX-2/prostaglandin pathway	Propranolol + etodolac for 35 days Propranolol: 20 mg BID → 80 mg BID on surgery day + next morning → 40 mg BID for 6.5 days → 20 mg BID for 22 days Etodolac (400 mg BID)	210	Unknown* Completion estimated: Jan 2026	Cancer recurrence up to 5 years Tumor and blood biomarkers
NCT04493489	Phase II, randomized, parallel-assignment, single-masked (participant)	Non-muscle-invasive bladder cancer, adjuvant treatment after TURBT with BCG	β-adrenergic signaling	Propranolol (10 mg TID, increased to 20 mg TID and then 40 mg BID) + BCG vs BCG alone	242	Unknown status (last update: July 2020) Completion estimated: Sep 2024	2-year recurrence-free survival
NCT04682158	Phase II, randomized, parallel-assignment, single-blind interventional	Esophageal adenocarcinoma undergoing neoadjuvant or definitive chemoradiation	β-adrenergic signaling	Propranolol (30 mg PO BID for 4–8 weeks) + paclitaxel/carboplatin with radiation	106	Recruiting Completion estimated: Apr 2029	Adverse events and progression-free survival up to 5 years Secondary endpoint: overall survival
NCT05741164	Phase II, single-arm, open-label, interventional	Metastatic/unresectable TNBC, checkpoint-inhibitor refractory	β2-adrenergic blockade	Propranolol + pembrolizumab	37	Recruiting Completion estimated: June 2029	Objective response rate within 6 months by irRECIST Secondary endpoints: 6-month PFS, OS, and safety
NCT03919461	Phase II, randomized, double-blind, placebo-controlled	Colorectal cancer, curative-intent surgery	β-adrenergic + COX-2/prostaglandin pathway	Propranolol + etodolac for 20 days perioperatively Propranolol: 20 mg BID → 80 mg BID on surgery day → 40 mg BID (week 1) → 20 mg BID (week 2) Etodolac (400 mg BID)	200	Recruiting Completion estimated: Feb 2027	5-year DFS Tumor and blood biomarkers
NCT05979818	Phase I, prospective, single-center, single-arm, open-label interventional	Stage IIIB–IIIC or IV metastatic NSCLC, EGFR/ALK/ROS1 driver-negative	β-adrenergic signaling	Propranolol hydrochloride (10 mg BID) + sintilimab (200 mg IV every 3 weeks) + platinum-based chemotherapy	6	Recruiting Completion estimated: Dec 2026	Objective response rate, disease control rate, and adverse events over 2 years Secondary endpoints: PFS, OS, and quality of life
NCT06839144	Phase II, randomized, quadruple-masked, placebo-controlled	Ovarian cancer, perioperative debulking surgery	β-adrenergic signaling + COX-2/prostaglandin signaling	Propranolol + etodolac	60	Recruiting Completion estimated: Aug 2026	Tumor and blood stress/inflammatory and pro-metastatic biomarkers Safety and adherence 3-year recurrence rate as secondary outcome
NCT07125391	Phase II, randomized, parallel-assignment, open-label, multicenter	Advanced high-grade serous ovarian cancer (FIGO stage III–IV)	β-adrenergic signaling	Propranolol (20 mg BID) + paclitaxel liposome + carboplatin for 3 neoadjuvant cycles, followed by interval debulking surgery	136	Recruiting Completion estimated: June 2027	Chemotherapy response score at approximately 3 months Pathological assessment of surgical specimens
NCT07311915	Phase (NA), single-arm, open-label interventional, multicenter	High-risk prostate cancer (high-risk/very high-risk or locally advanced, non-metastatic), neoadjuvant setting before radical prostatectomy	β-adrenergic signaling	Propranolol (10 mg TID, dose adjustment according to blood pressure) + novel endocrine therapy + androgen deprivation therapy	30	Not yet recruiting Completion estimated: Sep 2028	Pathological complete response and minimal residual disease at 12 weeks

*Unknown status: trial status has not been updated and remains unknown according to ClinicalTrials.gov.

ALK, anaplastic lymphoma kinase; BCG, Bacillus Calmette–Guérin; BID, twice daily; CRC, colorectal cancer; CT, computed tomography; DFS, disease-free survival; EGFR, epidermal growth factor receptor; FIGO, International Federation of Gynecology and Obstetrics; HCT, hematopoietic cell transplantation; irRECIST, immune-related Response Evaluation Criteria in Solid Tumors; IV, intravenous; NA, not applicable; NSCLC, non-small cell lung cancer; ORR, objective response rate; OS, overall survival; PFS, progression-free survival; PO, oral; QID, four times daily; RCT, randomized controlled trial; ROS1, ROS proto-oncogene 1; TID, three times daily; TNBC, triple-negative breast cancer; TURBT, transurethral resection of bladder tumor.

A randomized, double-blind trial (NCT00502684) evaluated perioperative propranolol plus etodolac in women undergoing primary breast cancer surgery to reduce surgery-induced immune suppression, stress/angiogenic signaling, and cancer recurrence. Published results by Sakis et al. (2026) reported pooled data from four RCTs involving 148 patients with breast, colorectal, and pancreatic cancers and found that perioperative propranolol plus etodolac was generally safe, with transient bradycardia occurring in 12% of treated patients versus 3% of placebo-treated patients, while no significant differences were observed in major postoperative adverse events. Notably, the regimen was associated with improved 8-year disease-free survival in colorectal cancer (2/15 vs. 9/18 events; *p* = 0.034), although this exploratory analysis was underpowered, supporting further evaluation of perioperative neural and inflammatory modulation as an anticancer strategy (46). A randomized, placebo-controlled biomarker trial with early-stage breast cancer cases (n = 38) evaluated 11 days of perioperative propranolol plus etodolac, initiated 5 days before surgery, to block catecholamine and prostaglandin signaling. The regimen was well tolerated and produced significant antitumor and immunomodulatory effects, including reduced epithelial-to-mesenchymal transition and activity of prometastatic/proinflammatory transcription factors (GATA-1, GATA-2, EGR3, and STAT3), decreased tumor-infiltrating monocytes, and increased tumor-infiltrating B cells. Treatment also prevented perioperative increases in IL-6 and C-reactive protein, prevented declines in stimulated IL-12 and IFN-γ production, reduced postoperative mobilization of CD16− classical monocytes, and increased CD11a expression on circulating NK cells (47). A complementary phase II biomarker trial in early-stage breast cancer (n = 38) similarly evaluated 11 days of perioperative propranolol plus etodolac, initiated 5 days before surgery. The treatment was well tolerated and reduced systemic and tumor-associated pro-inflammatory/pro-metastatic signaling, including IL-6, CRP, IFN-γ, TRAIL, NF-κB, STAT3, and ISRE activity, while reducing tumor Ki-67 expression and favorably modulating PBMC transcription factors linked to poor prognosis, angiogenesis, EMT, and proliferation (48).

A randomized, double-blind, placebo-controlled biomarker trial in 34 patients with colorectal cancer evaluated 20 days of perioperative propranolol plus etodolac, initiated 5 days before surgery. Treatment was well tolerated and reduced epithelial-to-mesenchymal transition and CD14+ monocyte infiltration, increased CD56+ natural killer cell infiltration, and favorably modulated 12 of 19 CRC-related transcription factors, including GATA, STAT, EGR, and CREB families. Three-year recurrence was lower with treatment (12.5% vs. 33.3%, *p* = 0.239), with a stronger trend among protocol-compliant patients (0% vs. 29.4%, *p* = 0.054) (49). A phase II randomized, open-label pilot trial (NCT02420223) evaluated peri-HCT propranolol in 25 patients with multiple myeloma undergoing autologous hematopoietic stem cell transplantation. Propranolol, initiated before HCT and continued for 28 days, significantly reduced β-adrenergically mediated gene expression versus controls (*p* = 0.017), although engraftment, infection, and myeloma response did not differ significantly. In the associated publication, Knight et al. (2020) showed reduced CTRA-related gene expression and CD16− classical monocyte activation, with increased CD34+ and decreased CD33+ myeloid progenitor–associated transcripts, providing molecular evidence that peri-HCT β-adrenergic blockade can modulate stress-related pathways and supporting its further investigation as an adjunctive anticancer strategy (50).

## 4 Implications for drug delivery

As discussed above, preclinical evidence and emerging clinical trials indicate that tumor-associated nerves actively regulate tumor behavior and represent potentially actionable components of tumor–neural circuits. However, we argue that the therapeutic implications extend beyond systemic inhibition of individual neural pathways. If neuronal signaling is spatially organized within specific tumor niches, where a neuromodulatory drug is delivered may be as important as its molecular target. This raises a largely unexplored question: can drug-delivery systems selectively target tumor–nerve interfaces while minimizing exposure to normal neural tissues? We therefore propose a conceptual shift towards neuromodulatory drug-delivery systems that target functional communication between tumor cells and neurons rather than tumor cells alone. This approach can be developed around four complementary principles (1): molecular targeting (2), anatomical targeting (3), and (3) neuroimmune targeting, each addressing a different aspect.

Despite advances in tumor-targeted drug delivery, clinically translated nanocarriers remain largely focused on improving the accumulation and delivery of conventional cytotoxic agents within tumor tissue. For instance, already marketed products such as liposomal doxorubicin are available as injectable formulations (NDA 050718; ANDA 203263, 207228, 208657, 212219, 212299, 215178, and 219199), liposomal irinotecan (NDA 207793), and nanoparticle albumin-bound paclitaxel (NDA 021660), all of which exploit enhanced intratumoral drug accumulation. Similarly, prostate-specific membrane antigen-targeted nanoparticle BIND-014 (NCT01812746) delivers docetaxel with enhanced tumor uptake, demonstrating encouraging antitumor activity, prolonged radiographic progression-free survival in patients with metastatic castration-resistant prostate cancer (51). CRLX101, a cyclodextrin-based polymeric nanoparticle conjugated with camptothecin, demonstrated favorable safety, prolonged systemic drug exposure, and encouraging antitumor activity, with 64% of patients achieving stable disease and a median progression-free survival of 3.7 months at the maximum tolerated dose. Patients with non-SCLC achieved a 73% stable disease rate and a median progression-free survival of 4.4 months (52). However, these approaches primarily target the tumor mass and are not designed to specifically address the tumor–nerve interface, particularly the invasive and synaptically active regions where neuronal signaling may promote tumor progression.

### 4.1 Molecular targeting

Systemic administration of conventional neuroactive agents, including antiepileptics (53), and AMPA receptor antagonists (54), is constrained by dose-limiting neurocognitive toxicity. Targeted nanocarriers could localize neuromodulatory agents to tumor–nerve interfaces while minimizing systemic exposure and modulating glutamate receptor pathways, including GluA2-lacking AMPA receptors. In a stroke model, FSC231-loaded liposomes (256 nm, 58% encapsulation efficiency) effectively delivered a PICK1 inhibitor that inhibited the PICK1–GluA2 interaction and prevented OGD/R-induced degradation of the GluA2 AMPA receptor subunit. However, GluA2-lacking AMPA receptors are also expressed in normal neural tissues (55), therefore, receptor targeting alone is unlikely to provide sufficient specificity and should be combined with stimulus-responsive strategies. Supporting this, gold nanoparticles (20 nm) functionalized with the AMPAR ligand NBQX through a PEG6 linker (44 nm) selectively bound to AMPARs on primary human astrocytes (*in vitro*), achieving membrane labeling in nearly 100% of cells. Upon laser irradiation, the membrane-tethered nanoparticles induced photothermal depolarization (~73% increase in membrane-potential signal) without significant cytotoxicity (>96% cell viability) (31). However, it’s *in vivo* specificity and efficacy remains to be determined. Two further considerations bear on this route. The most promising molecular targets are those expressed by tumor cells at the tumor–nerve interface rather than by neurons themselves. These include postsynaptic GRIN2D in PDAC, whose genetic deletion or pharmacological inhibition reduced tumor burden, metastasis, and peritumoral innervation while prolonging survival in orthotopic models (25), NMDA and AMPA receptor subunits expressed by glioma and brain-metastatic cells (22, 24), and cancer cell-derived neuron-like cells, whose silencing inhibited tumor growth *in vivo* (37).

### 4.2 Anatomical targeting

Current evidence provides stronger support for anatomical targeting than for molecular targeting. Nanoparticles designed to target nerves offer a promising example. For instance, NP41 is a nerve-binding peptide identified through phage display that binds to laminin-421 and laminin-211, proteins in the extracellular matrix surrounding peripheral nerves. Experimental results confirmed its binding to these laminins, with laminin-421 identified as the primary target. NP41 also highlighted nerve structures after transection, even months after injury, when these structures were no longer visible to the naked eye (56). You H. et al. developed NP41-functionalized superparamagnetic iron oxide nanoparticles loaded with propranolol for both nerve imaging and targeted therapy in prostate cancer. In an orthotopic mouse model, these nanoparticles enabled sensitive and specific visualization of tumor-associated nerves using MRI and magnetic particle imaging. Fluorescence imaging further showed that NP41 accumulated in tumors, reaching a maximum tumor-to-normal tissue ratio of 6.1 ± 0.28, approximately three times that of free dye. Therapeutically, the formulation increased the mouse survival rate to 83.3% by day 45, compared with 46.9% for free propranolol, 42.9% for drug-free nanoparticles and 11.1% for the untreated control group. Treatment also reduced tumor growth, intratumoral nerve density and lymph-node metastasis. Importantly, no detectable impairment of peripheral nerve function or notable pathological changes in major organs were reported in the treated animals (32). To our knowledge, You H. et al. study represents the only published evidence of a nerve-ligand-directed carrier delivering a neuromodulatory drug to cancer-associated nerves with measurable antitumor effects.

Device-enabled regional delivery provides a second line of evidence, supported by clinical studies. In glioma, where neuron–tumor synapses are well characterized (16, 17, 20–22), low-intensity pulsed ultrasound (1 MHz, 11.5 mm device) with intravenous microbubbles has enabled transient blood–brain barrier (BBB) disruption and has been shown to be safe and repeatable in patients with recurrent glioblastoma (57, 58). In one study, 19 of 21 enrolled patients received ultrasound treatment, with BBB disruption visible on T1-weighted MRI in 52 of 65 sessions. Adverse events were manageable, and no carboplatin-related neurotoxicity was observed. Clear BBB disruption was associated with longer median progression-free (4.11 vs 2.73 months) and overall survival (12.94 vs 8.64 months), although the small cohort limits interpretation (58). In a recent Phase 1 trial, an implantable nine-emitter ultrasound device enabled repeated BBB opening increasing peritumoral concentrations of carboplatin 5.9-fold and albumin-bound paclitaxel 3.7-fold (p = 0.0001 and p < 0.0001, respectively). Across 68 treatment cycles, no progressive neurological deficits attributable to ultrasound were observed, and the main ultrasound-related adverse effect was transient grade 1–2 headache (71% of patients), supporting the feasibility of repeated, ultrasound-mediated drug delivery in recurrent glioblastoma. Further clinical evaluation of this study in an ongoing Phase 2 trial combining LIPU-MB with albumin-bound paclitaxel and carboplatin (NCT04528680) (59). A randomized, open-label Phase III trial (NCT05902169) is evaluating SonoCloud-9-mediated BBB opening combined with carboplatin against standard chemotherapy with lomustine or temozolomide in patients undergoing surgery for first recurrent glioblastoma. With an estimated enrolment of 560 patients, the trial aims to determine whether ultrasound-enhanced carboplatin delivery improves treatment efficacy, with completion anticipated in 2028. Two early-phase clinical trials (NCT03566199, NCT04264143) are evaluating convection-enhanced delivery of MTX110 (panobinostat nanoparticle formulation) directly into the brain to treat diffuse midline glioma, including diffuse intrinsic pontine glioma. This approach bypasses the BBB and enables targeted drug delivery to the tumor while potentially limiting systemic toxicity. It is particularly relevant because GABAergic and glutamatergic neuron–glioma synapses within the pons can promote tumor proliferation and growth.

### 4.3 Delivery to the neuro-immune axis

A careful review of the existing literature (Section 2.3) indicates that several promising therapeutic targets lie within neuroimmune pathways rather than directly within neuron–tumor interactions, with each potentially requiring a distinct delivery strategy. CGRP–RAMP1 signaling and nociceptor-derived IL-6 influence T cells and myeloid cells in both the tumor microenvironment and tumor-draining lymph nodes (35, 42–44). Therefore, delivery systems designed to reach lymph nodes may be important, as carriers that accumulate mainly in the primary tumor may fail to address these nodal immune responses. Cancer-induced nerve injury also activates ATF3-dependent transcriptional signaling in neuronal cell bodies, suggesting that effective intervention may require drug exposure in the dorsal root or trigeminal ganglia rather than only at nerve endings within the tumor (44). Retrograde axonal transport could therefore be explored as a potential route for delivering therapeutics to these neuronal sites. For example, a recent study demonstrated that gold nanoparticles conjugated with wheat germ agglutinin–horseradish peroxidase underwent more efficient retrograde transport along sensory neuron axons than unmodified gold nanoparticles. In a microfluidic neuronal model, the conjugated nanoparticles accumulated more rapidly in neuronal cell bodies and exhibited faster transport, with longer movement periods along the axon (60).

From a clinical perspective, an approach with greater immediate relevance may be to combine an approved neuromodulatory drug with immune checkpoint blockade at a dose sufficient to achieve local activity while limiting systemic exposure. Supporting this, a Phase I clinical trial in patients (n = 9) with treatment-naïve metastatic melanoma found that combining propranolol (10, 20, and 30 mg twice daily) with pembrolizumab (200 mg every 3 weeks) was well tolerated and showed promising antitumor activity, with an objective response rate of 78%. Responders also showed increased IFN-γ and decreased IL-6 levels, although these biomarker changes were not statistically significant (61). Clinical evidence further supports the relevance of neuroimmune modulation. Perioperative propranolol plus etodolac has demonstrated immunomodulatory effects across breast and colorectal cancer studies, with exploratory evidence of improved long-term disease-free survival in colorectal cancer (46–49). Similarly, peri-transplant propranolol reduced β-adrenergic stress-related gene expression in patients with multiple myeloma, although clinical outcomes were unchanged (50).

## 5 Limitations and challenges

In clinical practice, drugs such as propranolol and approaches such as ultrasound-mediated BBB opening and convection-enhanced delivery have shown promising clinical or translational potential. However, selectively modulating tumor–neural interactions remain challenging, particularly since the therapeutic window depends on disrupting pathological signaling while preserving normal neural function.

At the molecular level, GRIN2D deletion or inhibition reduced tumor burden, metastasis, and peritumoral innervation in PDAC models (25). However, GluN2D-containing NMDA receptors also regulate physiological neuronal activity, as evidenced by reduced neuronal excitability and burst firing following GluN2D ablation in reticular thalamic neurons, alongside altered inhibitory neurotransmission and seizure susceptibility (62). Additionally, GluA2-lacking AMPA receptors have been implicated in glioma–neuron interactions (22), while GluN2B-containing NMDA receptors have been associated with interactions between brain-metastatic cancer cells and neurons (24). Silencing cancer cell-derived neuron-like cells also inhibited tumor growth *in vivo* (37). However, these targets may not be restricted to tumor-associated tissues, and their expression and functional relevance require validation in human specimens.

At the anatomical level, targeted delivery may improve drug exposure at the interface but can also expose adjacent healthy nerves. NP41-functionalised superparamagnetic iron oxide nanoparticles carrying propranolol demonstrated nerve-associated targeting and antitumor activity in a prostate cancer mouse model (32). However, NP41 binds nerve-associated laminins without distinguishing tumor-associated nerves from healthy nerves (58). Similarly, ultrasound-mediated BBB opening has enhanced drug delivery in glioblastoma (59), while convection-enhanced delivery has enabled local drug administration in diffuse midline glioma, as investigated in clinical trials (NCT03566199 and NCT04264143). Although these approaches demonstrate the potential of regional delivery, selective modulation of tumor–neural signaling remains to be established.

At the neuroimmune level, targeting neurotransmitters, neuropeptides, and cytokines presents additional challenges since these mediators regulate both antitumor immunity and normal physiological functions. Clinical studies have explored propranolol with pembrolizumab in metastatic melanoma and perioperative propranolol plus etodolac in colorectal cancer, reporting preliminary efficacy signals and general safety (46, 61). Although encouraging, these findings do not establish efficacy, highlighting the need for comprehensive neurological safety and neurotoxicity assessments (63). Prespecified stopping criteria are also important for patients with pre-existing chemotherapy-induced peripheral neuropathy and when targeting pathways involved in cardiac, respiratory, or gastrointestinal function (64).

Most importantly, endpoints could be adapted to assess normal-nerve preservation. In the literature, animal studies of chemotherapy-induced peripheral neurotoxicity have assessed multiple aspects. For instance, animal studies of cisplatin- and paclitaxel-induced neurotoxicity have combined serial serum neurofilament light chain (NfL) measurements with nerve conduction studies, morphological assessments, and intraepidermal nerve fiber density analysis to correlate NfL levels with the onset and severity of axonal damage (63). Oxaliplatin- and paclitaxel-induced peripheral neuropathy has been assessed using cold sensitivity and mechanical allodynia tests, nerve conduction and excitability studies, caudal nerve morphometry, and intraepidermal nerve fiber density analysis (65). Doxorubicin- and cyclophosphamide-induced cognitive effects have been investigated through behavioral assessments of learning, memory, motor coordination, grip strength, anxiety-like behavior, object recognition, and olfactory function, alongside hippocampal dendritic spine morphology, antioxidant status, and inflammatory cytokine levels (66). In a clinical study of paclitaxel-induced peripheral neuropathy, NfL release from human induced pluripotent stem cell-derived sensory neurons and serial serum NfL measurements in patients with ovarian cancer were investigated as indicators of neurotoxicity and predictors of neuropathy-related treatment discontinuation, dose reduction, and severe neuropathy (67). These endpoints provide a basis for evaluating neurotoxicity and assessing normal-nerve preservation in tumor–nerve-targeted delivery studies.

## 6 Conclusion

The available literature has fundamentally reshaped our understanding of neuro-oncology, positioning the nervous system not as a passive bystander but as an active regulator of cancer biology. Our central argument is that clinical studies of propranolol, etodolac, and botulinum toxin A, conducted predominantly using systemic administration, demonstrate the therapeutic relevance of neural signaling in cancer, while drug-delivery strategies have not yet fully adapted to this emerging paradigm. Other agents, including riluzole, propranolol, NBQX, and glutamate-receptor antagonists, further illustrate potential pharmacological approaches to modulating tumor–neural signaling, although their evidence and clinical maturity vary. Available approaches predominantly target tumor cells, whereas emerging evidence suggests that critical regulatory signals may reside at tumor–nerve interfaces. Although propranolol-conjugated NP41 nanoparticles provide proof of concept for nerve-directed drug delivery in prostate cancer, validation across tumor types and systematic assessment remain necessary. We argue that this hypothesis is now sufficiently defined to be experimentally tested and potentially falsified. This framework might provide a rationale for developing and evaluating delivery systems designed to selectively modulate tumor–neural communication. This framework provides a rationale for evaluating delivery systems designed to selectively modulate tumor–neural communication.
